# Phonosurgical Treatment of Laryngeal Leukoplakia and Dysplasia: Results of Multidimensional Voice Diagnostics Including the VEM

**DOI:** 10.3390/diagnostics16081242

**Published:** 2026-04-21

**Authors:** Moonef Alotaibi, Felix Caffier, Ahmad S. A. Alghamdi, Carla Azar, Martin Kampmann, Tadeus Nawka, Dirk Mürbe, Philipp P. Caffier

**Affiliations:** 1 Department of Audiology and Phoniatrics, Charité–Universitätsmedizin Berlin, Corporate Member of Freie Universität Berlin, Humboldt-Universität zu Berlin, and Berlin Institute of Health, Campus Charité Mitte, Charitéplatz 1, 10117 Berlin, Germany; alotaibi.moonef@gmail.com (M.A.); ahmad-saeed.alghamdi@charite.de (A.S.A.A.); carla.azar@charite.de (C.A.); martin.kampmann@charite.de (M.K.); tadeus.nawka@charite.de (T.N.); dirk.muerbe@charite.de (D.M.); 2 Department of Otorhinolaryngology, Head and Neck Surgery, King Fahad Medical City, P.O. Box. 59046, Riyadh 11525, Saudi Arabia; 3 SOMNOmedics, Am Sonnenstuhl 63, 97236 Randersacker, Germany; broozar@web.de; 4 Berlin Center for Musicians Medicine, Charité–Universitätsmedizin Berlin, Campus Charité Mitte, Charitéplatz 1, 10117 Berlin, Germany

**Keywords:** laryngeal leukoplakia, laryngeal dysplasia, precancerous lesions, microlaryngoscopy, laryngeal surgery, phonosurgery, transoral laser microsurgery, voice diagnostics, vocal outcomes, Vocal Extent Measure (VEM)

## Abstract

**Background/Objectives**: Laryngeal leukoplakia and dysplasia carry a variable risk of malignant transformation. Although microlaryngoscopic excision is standard of care, data on voice function are limited. Multidimensional diagnostics, including the Vocal Extent Measure (VEM), were employed to assess pre- and postoperative status while identifying factors associated with vocal outcomes. **Methods**: This retrospective cohort included 44 patients with histologically confirmed vocal fold leukoplakia or dysplasia. All underwent cold steel or laser-assisted phonomicrosurgery. Voice assessments were conducted pre- and three months postoperatively, comprising videolaryngostroboscopy, auditory-perceptual evaluation of grade, roughness and breathiness (GRB), self-assessment (Voice Handicap Index, VHI-9i), and objective acoustic-aerodynamic measures. **Results**: Overall, 57% of patients were active smokers; 73% consumed alcohol. Lesions were mostly unilateral (77%), craniomedially localized (65%), and involved up to one-third of the vocal fold (48%), with impaired mucosal wave (76%). Histopathology revealed mainly hyperkeratosis (52%) and dysplasia (35%). Recurrence rate was 14%, with histology unchanged. Postoperatively, subjective measures showed significant improvements (post- vs. preoperative), with decreased VHI-9i scores (10 vs. 14) and GRB ratings (*p* < 0.05). Objective measures showed positive trends, including enhanced vocal capacity (VEM 85 vs. 82), stability (jitter 0.6 vs. 0.8%), and aerodynamics (maximum phonation time 18 vs. 15 s). Phonosurgical method, histopathology, and age did not significantly affect voice outcomes; however, higher dysplasia grades and younger age showed trends toward greater VEM gains. **Conclusions**: Phonomicrosurgical excision of laryngeal leukoplakia and dysplasia effectively preserves or enhances vocal function. The VEM provides a reliable, quantitative complement to established voice diagnostics and should be integrated into standardized assessment protocols.

## 1. Introduction

The “Precancerous lesions” denote histopathological changes with the potential for malignant transformation. Within the larynx, such lesions are typically classified as laryngeal leukoplakia and laryngeal dysplasia. Epidemiological data indicate an annual incidence of approximately 10.2 per 100,000 men and 2.1 per 100,000 women presenting with newly diagnosed precancerous laryngeal lesions in the United States [1]. Among these, dysplastic leukoplakia carries a significantly increased likelihood of malignant transformation, which is notably higher in the larynx than in the oral cavity [2]. Multiple etiological factors may contribute to this progression, including tobacco use and alcohol consumption, which are linked to more than 75% of head and neck cancers [3]. Additional considerations include genetic variations and environmental issues like asbestos exposure [4]. Histopathological lesions associated with human papillomavirus (HPV) infection are also recognized as precancerous entities. Based on their oncogenic potential, HPV genotypes are classified into high-, intermediate-, and low-risk categories. Low-risk HPV types 6 and 11 are the main causes of recurrent laryngeal papillomatosis. In this condition, the reported rate of malignant transformation ranges from 1% to 7%, while the prevalence of dysplasia varies between 5 and 28% [5,6,7,8]. Furthermore, a retrospective study from Germany demonstrated the influence of advanced age and gender [9]: Patient groups aged >65 and 50–65 years had a higher risk of being diagnosed with laryngeal cancer (OR = 4.90 and 2.55, respectively). A clear male predominance was observed, with men showing a substantially greater likelihood of developing laryngeal cancer compared to women (OR = 4.09).

Laryngeal dysplasia classification was introduced over 60 years ago and has since been revised multiple times to standardize terminology and histopathological grading of squamous intraepithelial lesions [10]. The nomenclature is diverse and includes the terms “proliferative squamous cell lesions” [11], “epithelial hyperplastic laryngeal lesions” (EHLL) [12], “carcinoma in situ” (CIS) [13], “squamous intraepithelial neoplasia” (SIN) [14,15] “dysplasia, carcinoma in situ” [16], “intraepithelial neoplasia of the larynx” [17], “laryngeal intraepithelial neoplasia” (LIN) [18], and “squamous intraepithelial lesion” (SIL) [19]. The application of dysplasia classification systems varies across institutions; however, a commonly used system distinguishes the following lesion types: squamous hyperplasia (without atypia), SIL I (mild dysplasia), SIL II (moderate dysplasia), and SIL III (severe dysplasia/CIS). Sakr et al. discovered that the basement membrane remains clear and continuous in SIL I and SIL II, whereas in SIL III, it becomes thinned and occasionally discontinuous [20]. In the latter, the basement membrane has not yet been penetrated, thus the tumor is not growing invasively. The current WHO classification streamlines the four- and three-tier systems into two categories: low-grade squamous intraepithelial lesions (LSIL, including squamous hyperplasia and SIL I) vs. high-grade squamous intraepithelial lesions (HSIL, including–with regard to dysplasia–SIL II and SIL III) [21,22].

In diagnostic microlaryngoscopy, small glottal findings with suspected precursor lesions, CIS and T1a carcinomas can be completely removed via excision biopsy [23,24]. In this respect, the quality of life depends primarily on the voice quality and therefore on the extent of the resected vocal fold (VF) tissue, in addition to the state of health [25,26,27]. A thorough evaluation would require comprehensive pre- and post-interventional voice diagnostics, analogous to the protocols employed in large phoniatric departments for the management of various laryngeal pathologies [28,29,30,31]. The minimum requirement for assessment and documentation of impaired vocal function is a recording of connected speech [32]. However, most studies on laryngeal SIL do not focus on functional results, but on histologic and oncologic outcomes [3,33,34,35]. Therefore, the main goal of this exploratory study was to investigate in detail the vocal status in patients with laryngeal leukoplakia and dysplasia before and after phonomicrosurgical treatment. We hypothesized that voice function can be preserved or improved after careful removal of these pathologies. This is the first study to assess pre- and postoperative voice function using multidimensional, specific objective and subjective parameters, including the recently established Vocal Extent Measure (VEM) based on the voice range profile (VRP) [36].

## 2. Materials and Methods

### 2.1. Study Design and Patients

**Figure 1:**
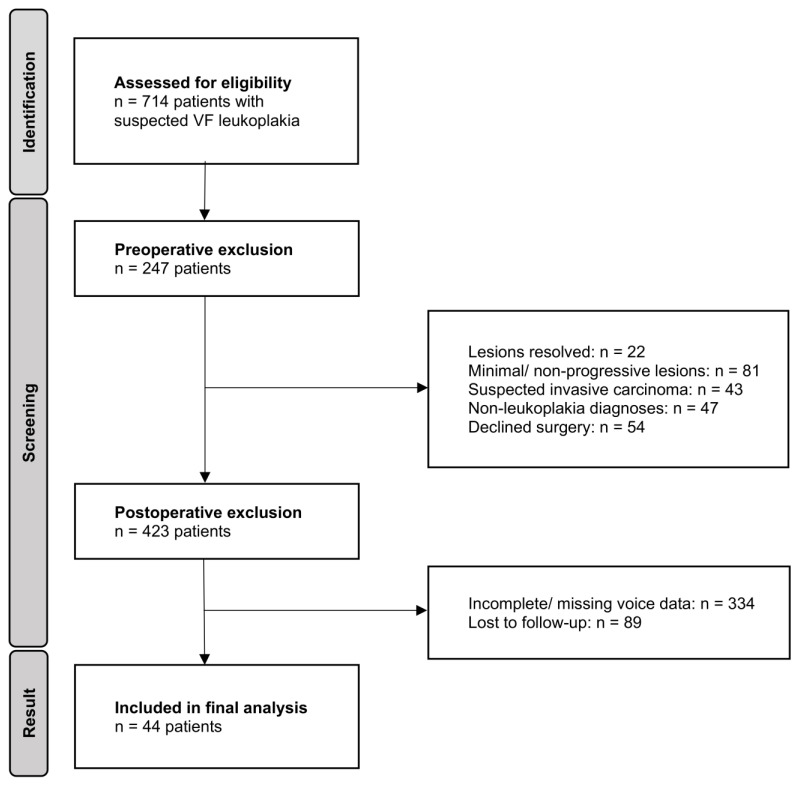
Flow diagram of patient selection. Study participants were sequentially excluded based on predefined clinical and methodological criteria. Exclusion categories were applied hierarchically, so that each patient was counted only once, corresponding to the first applicable criterion. Only individuals with complete pre- and postoperative multidimensional voice assessments and histopathological confirmation were included in the final analysis.

Over a 15-year investigation period, spanning from June 2009 to August 2024, a total of 714 adults presenting with voice symptoms and a referring ENT diagnosis of VF leukoplakia were retrospectively enrolled in this study. All patients were evaluated at the Department of Audiology and Phoniatrics, Charité–Universitätsmedizin Berlin, Germany, where clinical re-assessments were performed. A priori exclusion criteria comprised: (1) resolution of leukoplakic lesions at the time of presentation, (2) very small epithelial alterations demonstrating no progression or vibratory irregularities upon repeated videolaryngostroboscopy (VLS), (3) suspicion of more extensive malignant tumors (e.g., T1a squamous cell carcinoma), and (4) cases in which leukoplakia findings could not be confirmed (e.g., localized thick mucus, scars, cysts, papillomatosis, verruca vulgaris laryngis). A flow diagram illustrating patient selection and sequential application of exclusion criteria is shown in Figure 1. Data collection was performed preoperatively, during the phonosurgical procedure, and at routine follow-up visits. Vocal function was evaluated at least one day prior to transoral laryngeal microscopic surgery and three months following complete (in sano) resection and wound healing. All patients consenting to surgery with persistent leukoplakia-suspect VF lesions underwent direct microlaryngoscopy and surgical excision of the pathology under general anesthesia, using cold steel instruments, CO_2_ laser, or blue laser techniques. Comprehensive pre- and postoperative multidimensional voice assessments, as well as the presence of histopathological alterations, both benign and precancerous, were inclusion criteria. The study was conducted in accordance with the Declaration of Helsinki and approved by the local ethical review board (case no. EA4/196/23, approved on 7 November 2023).

### 2.2. Treatment Procedure and Follow-Up Protocol

The surgical equipment for endolaryngeal exposure included the Kleinsasser laryngoscope suspension system (Storz, Tuttlingen, Germany) and an OPMI Sensera operating microscope (Zeiss, Jena, Germany). Excisions were performed either with a cold steel dissection or laser-assisted. This involved the use of laryngeal scissors and forceps (Storz, Tuttlingen, Germany), the AcuPulse 30W/40 ST CO_2_ laser system (Lumenis, Yokneam, Israel), and the WOLF TrueBlue surgical laser (A.R.C. Laser, Nürnberg, Germany). Features of the CO_2_ laser (10,600 nm) comprised an applied output power range of 2–5 W, super-pulse mode, a spot size of 200 µm, and a focal length of 400 mm. The standard intraoperative safety protocols were followed, including the use of moist cloths to cover the patient, safety goggles, a laser endotracheal tube, and ventilation with an oxygen concentration below 30%. For the blue laser (445 nm), we used a 400 µm glass fiber in a staggered approach down to close-to-contact distances, with settings of 4–6 W, 26 ms pulse duration, and 300 ms pauses.

**Figure 2:**
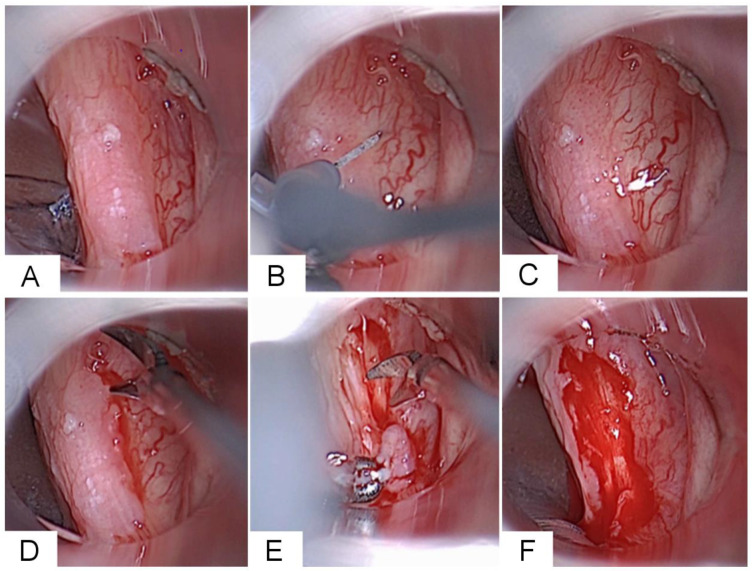
Intraoperative Surgical Technique for Resection of a Leukoplakic Vocal Fold (VF) Lesion Using Cold Steel Instruments. (**A**) Microlaryngoscopic exposure for inspection and palpation of the lesion on the right VF. (**B**) Hydrodissection with saline infusion. (**C**) Superficial epithelial layer completely separated from the underlying superficial lamina propria, ruling out an invasive lesion. (**D**) Microsurgical resection of the leukoplakia begins with an incision at the boundaries between healthy and diseased epithelium. (**E**) This is followed by careful stripping of the leukoplakia. (**F**) Wound directly after resection.

Following examination and palpation under the microscope, the VF was injected with saline containing epinephrine (1 mg/mL; 10 gtt in 10 mL NaCl) to assess the fixation of the lesion to deeper structures by expanding the epithelium. Suspicious lesions were excised at the point where they could be distinguished microscopically from the surrounding normal epithelium. Figure 2 presents the intraoperative surgical steps during microlaryngoscopic resection of a leukoplakic VF lesion in a patient example.

The specimens were sent for histopathological examination. Five board-certified, surgically experienced laryngologists performed the operations. Patients were observed on the ward for one or two nights postoperatively as a precautionary measure to monitor airway safety, manage potential postoperative bleeding, and provide immediate support for pain control and early complications. Everyone received vocal hygiene counseling before discharge. Voice rest after surgery was not advised. At discharge, all patients were given appointments at our clinic 2 weeks and 3 months postoperatively for follow-up examinations, including VLS and voice assessment. This 3-month interval allowed sufficient time for complete wound healing and functional recovery. Patients with longer-term follow-up underwent additional assessments either as part of routine reevaluation or in response to perceived changes in voice. In some cases, extended follow-up evaluations were performed annually to assess long-term outcomes and to examine whether postoperative voice improvements were maintained over time in the absence of recurrence.

### 2.3. Examiners’ Tools and Standards

The treatment outcome was examined using multidimensional voice function diagnostics, videolaryngostroboscopy (VLS) before and after surgery, and histopathological results following excisions. Rigid transoral or flexible transnasal endoscopes with integrated microphones (XION, Berlin, Germany) [37,38] were used for digital 2D or 3D VLS. The voice function was assessed subjectively and objectively via a computer-based approach.

Subjective measurement involved both the examiner’s auditory-perceptual evaluations and the examined person’s assessment of own vocal impairment. Voice samples were recorded live in the clinical setting and subsequently analyzed. Using the GRB system, an auditory-perceptual evaluation of recorded voice samples was carried out [32,39] on the basis of the standardized text “The north wind and the sun” (German version). Owing to its validated reliability, focus on the core perceptual dimensions of voice quality (grade, roughness, and breathiness), and well-established pathophysiological basis—with roughness reflecting aperiodic vocal fold vibration and breathiness reflecting incomplete glottal closure—the GRB scale provides an efficient and robust tool for auditory-perceptual assessment. The patient’s overall grade of hoarseness (G), roughness (R), and breathiness (B), as perceived by the examiner, were scored on a scale from 0 to 3 by two senior phoniatricians (0 = not existing, 1 = mild, 2 = moderate, 3 = severe). The average of the two independent GRB assessments was taken from each audio recording. To optimize evaluation objectivity, all pre- and postoperative audio recordings were shuffled and blinded regarding patient assignment and operative status. The 9-item voice handicap index (VHI-9i) was used to obtain a subjective vocal self-assessment [40] by answering the questions on a scale from 0 to 4 (0 = never, 1 = almost never, 2 = sometimes, 3 = almost always, 4 = always). Owing to its validated reliability and validity, reduced administration time, and comparable discriminatory power relative to the original VHI-30 and other longer short-form versions (e.g., VHI-12, VHI-10), the VHI-9i represents an efficient and clinically robust instrument for assessing patient-reported voice handicap. In a summarizing statement, patients had to assess their overall self-perceived vocal impairment (VHIs) at the present time of questioning on a scale from 0 to 3 (0 = normal, 1 = mild, 2 = moderate, 3 = severe), representing a single-item overall rating separate from the VHI-9i total score. The full VHI-9i and the VHIs are provided in Appendix A, along with relevant references.

The DiVAS software (v2.8; XION, Berlin, Germany) was used to conduct acoustic-aerodynamic analyses and VRP measurements to collect objective quantitative data on the speaking and singing voice. Recordings were performed in a sound-treated room (voice laboratory) of our department, with background noise levels below 40 dB(A), and a constant mouth-to-microphone distance of 30 cm maintained using a head-mounted microphone. This recording distance was selected to reduce proximity effects and plosive-related pressure artifacts while controlling for room reflections, ensuring reliable acoustic signal capture, and was supported by automatic SPL calibration within the system. Soft phonation threshold (SPLmin), highest and lowest pitch (F0max, F0min), maximum phonation time (MPT), jitter, dysphonia severity index (DSI) [41,42,43], and VEM [36] were among the parameters that were gathered. The DSI is a composite measure calculated as a weighted combination of F0max, SPLmin, MPT, and jitter, providing an overall estimate of dysphonia severity. As reported in the VRP, the VEM assesses the patient’s frequency range and dynamic performance. The vocal capacity is expressed as an interval-scaled value, typically ranging from 0 to 120. A high VEM characterizes a large VRP; conversely, a smaller VRP results in a lower VEM.

### 2.4. Data Processing and Analysis

Descriptive statistics were employed to summarize the quantitative characteristics of the dataset. For all subjective and objective parameters, minimum and maximum values, medians, first and third quartiles, means, and standard deviations (SD) were calculated. To visualize the distribution of pre- and postoperative measurements, graphical methods such as boxplots and histograms were utilized.

Given their suitability for both continuous and ordinal data, Spearman’s rank-order correlations (r_s_) were calculated to assess the strength and direction of associations between pre- and postoperative variables. The Wilcoxon signed-rank test was used to determine whether changes in vocal function parameters following treatment were statistically significant. Corresponding mean differences and 95% confidence intervals (CI) were computed to quantify these changes. Furthermore, Spearman’s rank-order correlation was used to explore relationships between therapeutic changes (delta (Δ) values, calculated as post-treatment minus pre-treatment measurements) in relation to clinical variables, including age, sex, lesion size, localization, and histopathological diagnosis.

Analyses were performed according to the respective unit of interest, explicitly clarifying the unit of analysis for each endpoint. Lesion-level analyses were applied when outcomes referred to individual VF lesions, whereas patient-level analyses were used for patient-based variables. In cases of bilateral lesions, no statistical adjustment for within-patient clustering was conducted. All statistical analyses and visualizations were performed using IBM SPSS Statistics, version 28.0.1.0 (IBM, Armonk, NY, USA). The significance threshold was set at α = 0.05. Significance levels were denoted as follows: *p*  <  0.05 (*), *p*  <  0.01 (**), and *p*  <  0.001 (***).

## 3. Results

### 3.1. Subject Demographics and Baseline Assessment

Forty-four patients in all fulfilled the inclusion criteria, 15 females (34%) and 29 males (66%). The mean age of all patients was 61 years, with 58 years (range, 37–81) for females and 63 years (range, 30–80) for males. Regarding tobacco consumption, 25 patients (57%) had an average of 24 packs per year (range, 5–40), and they were active smokers. Two other subjects stopped smoking prior to receiving their diagnosis, and 17 patients (39%) were non-smokers. Thirty-two subjects (73%) consumed alcohol: every day (n = 3), at least once a week (n = 23), or seldom (n = 6). A total of 12 patients (27%) abstained from alcohol.

Regarding laryngeal lesions, the VFs were affected unilaterally in 34 patients (77%) and bilaterally in 10 patients (23%). Among the 54 glottal findings observed, 28% were confined to the right VF, 35% to the left VF, and 37% on both sides. VLS assessment of lesion extent at initial presentation revealed that nearly half of the cases (48%) involved up to one-third of the VF, while 39% extended across two-thirds, and in only 7 cases, the lesion affected the entire VF (13%). Approximately two-thirds of the cases (65%) were localized craniomedially, involving the medial VF margin, followed by lesions extending caudally (22%). Notably, only a small subset (13%, n = 7) was confined exclusively to the cranial VF surface. During VLS, phonatory VF mobility was impaired in 41 cases (76%), including 33 lesions (61%) with reduced and 8 lesions (15%) with absent vibrations. In contrast, nearly one quarter of cases (24%, n = 13) exhibited preserved phonatory VF mobility and regular mucosal wave propagation during phonation. Relevant preoperative patient and lesion characteristics are shown in Table 1 (left side).

Subjective auditory-perceptual evaluation of patients’ voices was preoperatively categorized with a mean of G1.5 R1.3 B0.8 (range 0–3). The VHI-9i yielded an average score of 14 ± 9, indicating self-reported complaints within the upper range of mild impairment. The objective acoustic and aerodynamic measures similarly reflected mild to moderate dysfunction, as demonstrated by parameters such as VEM (82 ± 36), DSI (1.8 ± 2.1), jitter (0.8 ± 0.5), SPLmin (55 ± 9 dB(A)), and MPT (15 ± 8 s). Correlation analysis performed on preoperative values showed that VEM correlated with other objective parameters: strongly with DSI (rs = 0.75 ***) and SPLmin (rs = −0.63 ***), moderately with MPT (rs = 0.41 **), and weakly with jitter (rs = −0.34 *). Furthermore, VEM was also correlated with subjective parameters, i.e., moderately with G (rs = −0.58 ***), R (rs = −0.55 ***) and VHI-9i (rs = −0.47 **), and weakly with B (rs = −0.38 *) and VHIs (rs = −0.33 *).

### 3.2. Intraoperative Assessment

**Figure 3:**
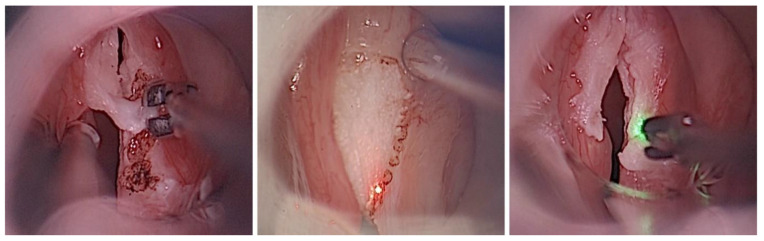
Intraoperative comparison of treatment modalities: Removal of leukoplakic vocal fold changes using cold steel forceps (**left**), CO_2_ laser (**center**), and blue laser (**right**).

Microlaryngoscopic removal was performed via cold steel instruments in 30 lesions (56%), while 24 findings (44%) were treated with laser-assisted phonosurgery. Among the latter, the CO_2_ laser was used in 18 lesions (33%), and the blue laser in 6 (11%). The choice of surgical modality was not determined by specific lesion characteristics, but rather by pragmatic and individualized factors, including the availability of laser systems (e.g., due to parallel use in other procedures), surgeon preference and experience, and, in selected cases, patient preference discussed during informed consent. Both approaches were considered technically equivalent for the intended subepithelial resections, and no bias was observed indicating that lesion size, localization, or morphological features influenced the selection of the surgical technique. In all of these approaches, following subepithelial infusion, the keratotic epithelium was carefully removed from the cranial VF surface (Figure 3). In cases of larger lesions surpassing the VF margin, excision was extended to the caudal regions. Deeper layers were spared, although the lamina propria could not always be preserved entirely, depending on the extent of the lesions. The use of the blue laser slightly extended the operative time but demonstrated a beneficial effect on hemostasis, exhibiting hemangiolytic properties. Regardless of the treatment modality, all leukoplakic lesions were successfully excised under microscopic visualization: according to the classification of endoscopic cordectomies of the European Laryngological Society (ELS), type I (subepithelial) resections with complete preservation of the lamina propria were performed in 46 VF lesions (85%), while type II (subligamental) resections including the superficial lamina propria were required in 8 lesions (15%).

### 3.3. Histopathological Results and Surgical Outcomes

Histopathological examination confirmed keratotic alterations characterized by hyperkeratosis and hyperparakeratosis in 52% of cases (28/54). Dysplasia was identified in 19 biopsies (35%), most frequently classified as SIL I (17%), followed by SIL III (13%) and least often SIL II (3 cases). Other diagnoses comprised four cases of reactive inflammation, along with solitary occurrences of fibrosis, sclerosis, and acanthosis (each n = 1). Primary surgical intervention resulted in the complete excision of leukoplakic lesions in 42 patients (96%). A second excision was necessary in 2 subjects, as a SIL III residuum could not be excluded (narrow margin vs. R1 status). In both cases, SIL III remnants were confirmed and completely resected during the second operation.

Within the mean postoperative observation period of 16 months (range, 3–46), 6 patients (14%) experienced lesion reappearances; follow-up duration varied and should be interpreted descriptively. Among them, 4 individuals initially diagnosed with SIL III presented with the same diagnosis upon recurrence, while 2 patients with keratosis also showed recurrence of keratosis. The average recurrence-free interval was 19 months (range, 3–40). Each patient experienced only a single relapse during follow-up. All recurrent laryngeal lesions were successfully treated via phonosurgery. Relevant postoperative patient and lesion characteristics are shown in Table 1 (right side).

**Figure 4:**
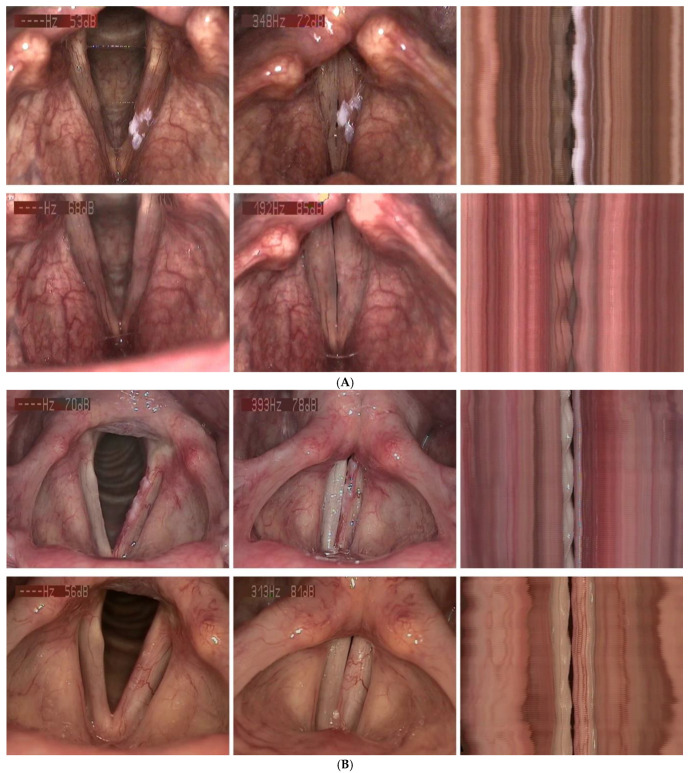

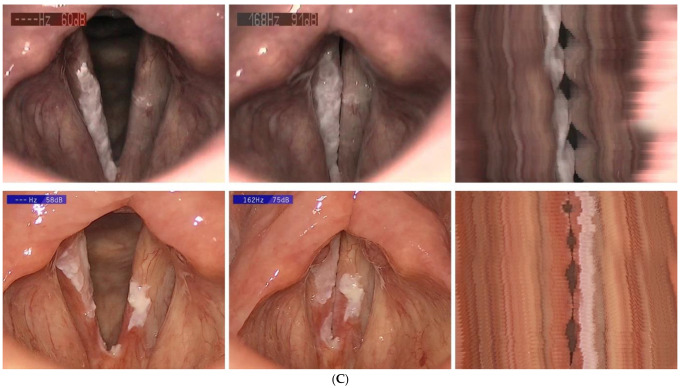
(**A**) Upper row: 59-year-old male patient with SIL II of the left glottis. Preoperatively, about one-third of the VF was affected, with reduced phonatory left VF mobility in videostrobokymography. Lower row: Three months after phonosurgery, the healing process finished, no signs of recurrence, complete glottal closure, and restored phonatory mobility (normalized, regular, and symmetric oscillations). (**B**). Upper row: 63-year-old female patient with SIL III of the left glottis. Preoperatively, about two-thirds of the VF were affected, with absent left VF mobility in videostrobokymography. Lower row: Three months after phonosurgery, the healing process finished, no signs of recurrence, complete glottal closure, and improved VF vibrations during phonation (stiffness-related reduced amplitude). (**C**) Upper row: 76-year-old male patient with hyperkeratosis of the glottis, right greater than left. Preoperatively, the entire right VF length was affected, but the phonatory VF mobility was good in videostrobokymography. Lower row: Three months after phonosurgery, the healing process finished, complete glottal closure, and hyperkeratosis recurrence on both sides, with continuing good vibration patterns during phonation.

The surgical procedures were performed without subsequent complications or impairments in respiratory or swallowing function. VLS check-ups displayed fibrin formation on the wound surfaces, followed by the production of healthy tissue during healing. While larger lesions, more extensive localizations, and HSIL histopathology were associated with more pronounced glottal tissue excision, smaller superficial findings showed stable epithelial regeneration on the preserved lamina propria without relevant defects or scarring. Among the more severely affected patients, postoperatively rigid VFs gradually regained phonatory mobility. Three months after intervention, vibratory function during phonation had improved significantly compared to the VLS baseline (*p* < 0.001): normal VF vibrations with regular mucosal wave propagation were observed in 41 patients (76%), reduced vibratory characteristics in 13 patients (24%), and no residual immobility was detected in any patient who underwent phonosurgery. Figure 4A–C gives impressions of the pre- and postoperative VLS findings with videostrobokymographic visualization of the VF oscillations.

### 3.4. Objective and Subjective Vocal Outcomes

Compared to the preoperative measurements, vocal function improved three months after phonosurgical excisions in most patients. Regarding objective parameters, these changes were statistically not significant. Apart from the DSI and F0max, they all showed slight improvements on average, including a higher vocal capacity and stability (VEM 85 ± 33; jitter 0.6 ± 0.3), a lower soft phonation threshold (SPLmin 54 ± 9 dB(A)), and a longer maximum phonation time (MPT 18 ± 22 s). Table 2 further quantifies the magnitude of changes by presenting mean differences with 95% CI between pre- and post-therapeutic values for all lesions and relevant pathology groups. The underlying histopathological diagnosis did not significantly influence vocal outcomes. However, higher initial dysplasia grades were associated with greater numerical improvements in postoperative vocal capacity (VEM) and aerodynamics (MPT). Additionally, patients with SIL III lesions showed slightly more pronounced subjective breathiness (B).

**Figure 5:**
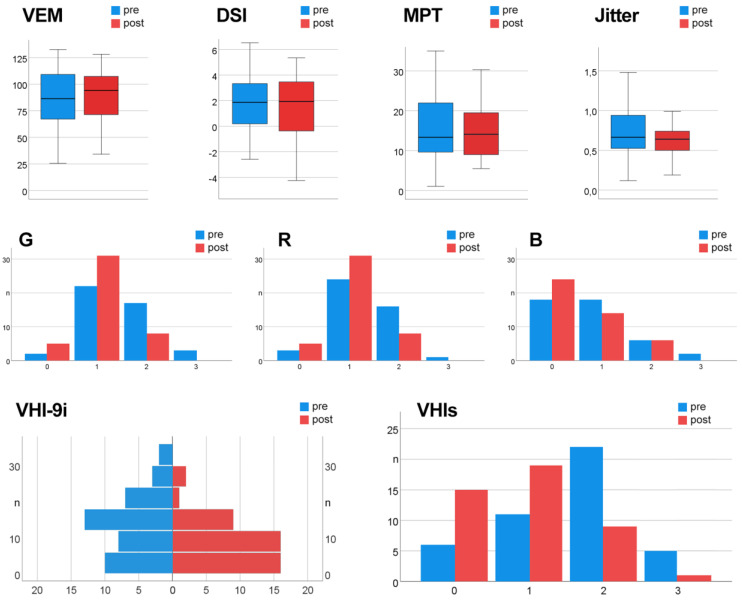
Comparison of established vocal measures before and three months after excision of laryngeal leukoplakia and dysplasia. Upper row: Objective acoustic and aerodynamic parameters, namely Vocal Extent Measure (VEM), Dysphonia Severity Index (DSI), jitter, and Maximum Phonation Time (MPT). Middle row: Subjective clinician-rated auditory-perceptual evaluation using the GRB classification (G—overall grade of hoarseness, R—roughness, B—breathiness). Lower row: Subjective self-assessed voice outcomes based on the 9-item voice handicap index (VHI-9i) and overall self-perceived vocal impairment (VHIs) scores.

Concerning post- vs. pre-treatment comparison of subjective parameters, auditory-perceptual GRB evaluation revealed that the voices were less hoarse (1.1 ± 0.6 vs. 1.5 ± 0.7), rough (1.1 ± 0.5 vs. 1.3 ± 0.6), and breathy (0.6 ± 0.7 vs. 0.8 ± 0.8). The subjective vocal self-assessment using the VHI-9i questionnaire showed a mean reduction of complaints by 4 points (10 ± 8 vs. 14 ± 9). The VHIs criterion indicated a change from moderately (2 ± 1) to mildly disturbed voices (1 ± 1). Improvements in all subjective parameters were significant (*p* < 0.05). Figure 5 gives an overview of selected vocal parameters assessed pre- and postoperatively. The boxplots display the median, quartiles, and the range of values of the objective data, highlighting the VEM increase. The histograms depict the distribution of subjective ratings, indicating a shift towards lower degrees of perceived severity.

Correlation analysis performed on postoperative values confirmed the strong relationship of VEM with DSI (rs = 0.64 ***) and SPLmin (rs = −0.71 ***). In addition, VEM correlated moderately with B (rs = −0.46 **) and F0max (rs = 0.40 **), and weakly with G (rs = −0.34 *). Further evaluation of potential influencing factors revealed that neither the specific therapeutic approach (cold steel vs. laser) nor age had any significant impact on treatment outcomes. However, for the objective parameters VEM and DSI, a trend was observed indicating that younger age was associated with greater postoperative vocal improvement (*p* = 0.095 and *p* = 0.072, respectively). Gender had little influence on vocal measures; however, a significant postoperative reduction in jitter was evident in females, but not in males (*p* < 0.05). A trend was also noted regarding lesion size: at initial presentation, women more frequently exhibited smaller findings than men (*p* = 0.085). Both the size and localization of the leukoplakic lesions had a significant impact on subjective vocal impairment as measured by the VHIs: the larger the affected VF area and the more pronounced the medio-caudal lesion extension, the greater the subjective postoperative voice improvement (*p* < 0.05 and *p* < 0.01, respectively). In contrast, lesion size and localization had no relevant effect on objective vocal parameters, except for a significant MPT increase when the entire VF length was initially involved (*p* < 0.05). In patients with extended follow-up, voice outcomes remained stable over time.

## 4. Discussion

Given the well-established success of surgical treatment for laryngeal leukoplakia and dysplasia, functional outcomes–particularly voice quality–should be considered an equally important therapeutic objective. In this study, vocal status was comprehensively assessed before and after phonomicrosurgical intervention. To evaluate phonatory outcomes, we employed a range of objective and subjective parameters, including the VEM derived from the VRP. For decades, VRPs have been collected in a standardized manner to assess individual vocal performance with respect to pitch (F0) and dynamic range [44,45,46]. However, traditional analysis of VRPs is primarily descriptive and relies heavily on visual interpretation and a limited number of exposed values. To overcome these limitations, the VEM was introduced as a novel, objective parameter for quantifying vocal capacity and classifying vocal performance [36]. It enables a more precise numerical evaluation of vocal function based on the VRP [28,47]. Unlike the established DSI, which expresses dysphonia as a negative criterion, the VEM was developed as a positive indicator of voice function [48]. Our hypothesis–that voice function can be preserved or even improved following careful surgical removal of laryngeal SILs–was confirmed.

### 4.1. Subjective and Objective Voice Outcomes

When comparing pre- and postoperative measurements, subjective and objective parameters yielded differing results. While all subjective and most objective parameters–except for the DSI and F0max–demonstrated improvements, statistically significant changes were observed only in the subjective measures. These results align with findings in the literature, where subjective evaluation, particularly the examiner’s auditory-perceptual judgment of the patient’s voice, is often considered the gold standard for voice assessment in clinical practice [28]. However, the human voice is a highly complex phenomenon that should be evaluated through a multidimensional approach [49,50,51]. According to European, American, and Asian guidelines, objective acoustic measurements represent an essential pillar of comprehensive voice diagnostics [52,53,54,55]. Nevertheless, their implementation in routine clinical settings is limited, often due to time constraints, and their value is frequently underestimated. In a recent review by Miri et al. on outcomes of office-based surgery for VF dysplasia and leukoplakia, only one of the 14 included studies incorporated a truly multidimensional voice assessment combining both subjective and objective parameters [35]. In that study, Hamdan et al. demonstrated significant postoperative improvements in jitter, shimmer, and MPT nine months after blue laser treatment in a cohort of 12 patients [56].

Postoperatively, the DSI showed a wider interquartile range than preoperatively, reflecting greater variability in composite outcomes, whereas VEM, MPT, and jitter became more uniform. This likely stems from the DSI’s composite nature: its constituent parameters F0max, jitter, MPT, and SPLmin were affected to differing extents across patients. F0max showed no improvement, probably because many patients already had relatively preserved phonatory function at baseline (VLS in 24% with normal phonatory mobility) and, in larger lesions, partial involvement of the superficial lamina propria may limit maximal VF tension. Consequently, no gains in F0max and minor changes in the other parameters constrained overall DSI improvement. Additional factors, including lesion extent, resection depth (ELS type I vs. type II), and individual healing processes, likely contributed to the broader distribution. Clinically, this underscores that while overall vocal function improved, the magnitude of change in composite indices like the DSI varies considerably, highlighting the value of multidimensional assessment.

Although our main postoperative assessment was at three months, extended follow-up in a subset of patients indicates that voice improvements are maintained over time. Beyond statistical significance, these improvements are clinically meaningful, reflected in lower VHI-9i scores, improved GRB ratings, and enhanced VEM, reflecting perceptible benefits in everyday voice use.

### 4.2. Role of VEM and Complementary Measures

For objective voice diagnostics, the novel VEM is particularly recommended, as it complements the established DSI and reliably reflects overall vocal performance [48]. The VEM captures vocal capacity as documented in the VRP of both healthy individuals and patients with various organic and functional voice disorders [28,29,30,31,47,48,57]. The severity of vocal impairment can be quantitatively assessed using a concrete numerical value, with additional categorization into quartiles to facilitate interpretation (Q1: VEM < 69, Q2: VEM 69 to < 93, Q3: VEM 93 to < 108, Q4: VEM ≥ 108). As such, the VEM represents a valuable extension of traditional voice assessment tools, clearly suitable for evaluating vocal function and for therapy monitoring in patients with VF dysplasia and leukoplakia. In this context, we observed significant correlations between the VEM and other vocal parameters. Preoperatively, these correlations were strong with the DSI and moderate with the VHI-9i, H, and R. Although therapy altered the strength of these associations, the VEM most accurately reflects the subjective evaluations of both voice experts (GRB rating) and patients (VHI-9i self-assessment).

According to our results, a trend was observed for both VEM and DSI, suggesting that younger patients experience greater postoperative vocal improvement, likely due to higher functional reserves and enhanced tissue regeneration associated with youth [58,59,60]. Additionally, a trend was detected regarding lesion size at initial presentation: women more frequently exhibited smaller lesions than men. This finding supports existing evidence of gender-based differences in sensitivity to vocal changes, the reporting of voice disorders, and subsequent engagement with healthcare services [61,62]. Lesion size and localization significantly influenced the perceived vocal handicap, yet they exerted no substantial effect on objective vocal parameters–except for a marked MPT increase following lesion removal when the entire VF length was initially affected. These findings confirm the clinical impression that VEM, DSI, MPT, VHI, and GRB capture distinct dimensions of vocal function and provide complementary objective and subjective assessments for evaluating vocal performance, voice impairment, phonation efficiency, vocal handicap, and voice quality.

### 4.3. Surgical Management and Functional Recovery

Therapeutically, surgical excision and histopathological examination represent the gold standard for assessing both the dignity and grade of leukoplakic VF lesions. Patients with persisting hoarseness typically undergo in-office VLS as an initial diagnostic step to identify the organic correlate and evaluate mucosal vibratory capacity, which may aid in predicting the presence of infiltrative lesions [63,64,65]. Reduced or absent phonatory VF mobility and suppressed mucosal wave propagation provide essential indications of invasive tissue growth [37,66]. However, neither diagnosis nor the decision to proceed with microlaryngoscopy and (excisional) biopsy should be based solely on stroboscopic findings, as factors such as the precise lesion location can significantly affect the observed degree of mucosal vibration changes [67,68]. Three-dimensional VLS, as developed and implemented in our center, appears to offer enhanced visualization and more detailed assessments, thereby facilitating evaluation [38,57]. Nonetheless, we generally recommend surgical excision for persistent leukoplakias, particularly when VLS reveals impaired VF mobility during phonation. In patients with histologically confirmed dysplasia, esp. HSILs, we provide close, structured long-term follow-up, supported by VLS. This management strategy aligns with the protocols employed by other established centers [34].

Timely detection of laryngeal neoplasms and their recurrences not only reduces surgical trauma, morbidity, and mortality but also improves therapeutic outcomes [69], such as minimizing postoperative glottal defects and thereby preserving voice quality. In our study, larger lesions, broader anatomical involvement, and HSIL histopathology were associated with more pronounced glottal tissue excision. In contrast, smaller and more superficial lesions exhibited stable epithelial regeneration over the preserved lamina propria, without notable scarring or structural defects. Interestingly, in patients with more extensive involvement, initially rigid VFs gradually regained phonatory mobility. At the three-month follow-up, vibratory function during phonation had significantly improved compared to the baseline VLS findings, suggesting functional recovery even in more severely affected cases. These observations are consistent with reports in the literature and with our own clinical experience of the healing process following transoral CO_2_ laser microsurgery for T1a glottic cancer [57].

### 4.4. Histopathology, Malignant Transformation, and Prognosis

The diagnosis of laryngeal dysplasia significantly influences both therapeutic decision-making and the extent of surgical resection. While the current WHO classification into two categories (LSIL and HSIL) provides a simplified framework for both pathologists and surgeons, interobserver variability and differences in treatment approaches remain [10], potentially impacting postoperative voice outcomes. Greater surgical radicality often results from concerns about recurrence and progression to malignancy. The risk of malignant transformation has been addressed in several studies and appears to correlate with the severity of dysplasia [3,70]. Isenberg et al. evaluated the risk of leukoplakia transformation using institutional data combined with a meta-analysis [71]. They reported an overall cancer development rate of 8.2%. Specifically, the risk of malignant progression was 3.7% in leukoplakia without dysplasia, 10.1% in mild/moderate dysplasia, and 18.1% in severe dysplasia/CIS. A systematic review of case series and meta-analysis by Weller et al. [72] revealed an overall malignant transformation rate of 14% over an average period of 5.8 years. The risk tripled with increasing dysplasia severity (10.6% in mild/moderate dysplasia vs. 30.4% in severe dysplasia/CIS).

In addition to histological grading, treatment modalities and significant risk factors must be considered when evaluating the likelihood and timeframe of malignant transformation. This complexity is reflected in the wide range of transformation rates reported across seven studies, summarized by van Hulst et al. [73]: 0–41.7% for mild dysplasia, 0–48% for moderate dysplasia, and 14.3–44.4% for severe dysplasia. A large retrospective study of 1444 patients with laryngeal SILs by Gale et al. [19] demonstrated a highly significant difference in malignant progression between low- and high-grade dysplasia, with rates ranging from 1.6% to 12.5%. A recent study by Mueller-Diesing et al. [74] reported comparably high malignant transformation rates for moderate and severe dysplasia (41% vs. 43%), supporting the updated classification of moderate dysplasia within the HSIL group. In our cohort, 14% of the patients experienced lesion recurrence, but none had histopathological progression. All individuals initially diagnosed with SIL III presented with the same grading upon return of the lesion, and all patients with keratosis demonstrated reappearance of keratosis. The mean recurrence-free interval was 19 months, and all recurrent laryngeal lesions were successfully managed with phonosurgical treatment.

As intended and supported by the literature [57,75,76], direct microlaryngoscopy under general anesthesia served both diagnostic and therapeutic purposes. In addition to inspection and palpation, hydrodissection was successfully employed in all patients to evaluate mucosal properties, including mobility and the extent of leukoplakia fixation to deeper tissue layers. Injection of saline with epinephrine enabled the separation of superficial epithelial leukoplakic lesions from the underlying superficial lamina propria. As described by Kono et al. [77], the benefit of this technique was limited in anatomical regions with minimal epithelial stretch, such as the anterior commissure and vocal process. However, the cutting characteristics of the CO_2_ laser and cold instruments differ between healthy and pathological tissue. Therefore, the combination of inspection, palpation, hydrodissection, and assessment of tissue response to instrumentation allowed for safe and complete, tissue-sparing excision of the lesions, even in challenging anatomical locations.

In our study, the underlying histopathological diagnosis did not show a statistically significant impact on overall vocal outcomes. However, individuals with higher initial dysplasia grades exhibited larger numerical enhancements in postoperative vocal capacity (VEM), aerodynamic function (MPT), and breathiness (B). These findings suggest that HSIL patients may benefit from greater improvements in voice quality, potentially because preoperative impairment allows measurable recovery after surgery. In contrast, patients with LSIL showed minimal postoperative change, possibly due to their better baseline voice function. This differential response highlights the importance of considering both baseline voice quality and lesion severity when evaluating surgical outcomes. In cases of advanced dysplasia, a ‘room for improvement’ effect may lead to more significant perceived benefits. Conversely, in mild lesions with near-normal preoperative voice quality, even minor structural changes from surgery could result in measurable or perceived vocal decline. Future studies should further investigate the mechanisms behind these outcome patterns. We believe that evidence-based treatment and effective voice preservation require standardized, multiparametric vocal assessment both pre- and postoperatively, ideally conducted in accordance with internationally established protocols, including the VEM measure.

### 4.5. Limitations and Future Directions

This study has several limitations, particularly the relatively small sample size, which limits statistical power and generalizability. In addition, the final analysis included only 44 of 714 initially screened patients, introducing potential selection bias. Second, the retrospective design precluded full control of confounding factors such as smoking and alcohol use. Third, treatment heterogeneity may limit intra-group comparisons; however, this reflects real-world practice, and vocal outcomes seem to depend more on pathology grade and lesion extent than on treatment modality. Fourth, follow-up duration varied and may have been too short to capture late recurrences or long-term vocal changes. In addition, follow-up beyond the scheduled postoperative visits was not protocol-driven but patient-initiated, which may have led to incomplete detection of recurrence events. Consequently, time-to-event data should be interpreted descriptively, and summary measures such as mean follow-up or recurrence-free intervals are subject to inherent limitations. Fifth, minor variability among surgeons could have influenced functional outcomes. Sixth, postoperative voice assessment was restricted to three months, preventing formal evaluation of long-term adaptation. Although some patients underwent extended follow-up assessments, these were not systematically collected, limiting the ability to draw robust conclusions on long-term voice trajectories despite indications of sustained functional stability. Seventh, some analyses were conducted at lesion level, while others were performed at patient level. In cases of bilateral lesions within a single patient, statistical independence cannot be fully assumed, which may introduce bias in lesion-based analyses. As the study was not designed for clustered or hierarchical modeling, this limitation should be considered when interpreting the findings. Eighth, the study was conducted at a single center, limiting generalizability to other settings. Ninth, no adjustment was made for multiple testing, which may increase the risk of type I error. Finally, despite comprehensive multidimensional diagnostics, both subjective ratings and acoustic measures remain susceptible to observer and environmental bias. Future prospective studies with larger, standardized cohorts and extended follow-up are needed to confirm these results and better define long-term vocal outcomes after phonomicrosurgery for laryngeal leukoplakia and dysplasia.

## 5. Conclusions

This study confirms that phonomicrosurgical excision of VF leukoplakia and dysplasia can preserve or even enhance vocal function, particularly when guided by comprehensive, multidimensional voice assessment. While subjective measures showed statistically significant postoperative improvement, objective parameters such as VEM and DSI also revealed positive trends, particularly in younger patients and those with more severe preoperative impairment. The VEM proved to be a valuable, complementary tool for quantifying vocal capacity and monitoring therapy outcomes, and it correlated well with both expert and patient evaluations. Although lesion size, location, and histopathological grading influenced surgical extent and recovery patterns, they did not significantly affect overall vocal outcomes, with HSIL patients often demonstrating the most marked postoperative improvements. These findings support the routine use of standardized, multiparametric voice diagnostics including the VEM for both pre- and postoperative evaluation, to ensure optimal voice preservation alongside effective treatment of laryngeal SILs.

## Figures and Tables

**Table 1:** Patient characteristics (*n* = 44) before (left) and after (right) microlaryngoscopic resection of laryngeal leukoplakia and dysplasia. Unless otherwise specified, data are expressed as number of lesions (total *n* = 54) and corresponding percentages within the group.

	Number	%		Number	%
Gender			Resection type		
male patients	29	65.9%	cold steel dissection	30	55.6%
female patients	15	34.1%	CO_2_ laser assisted	18	33.3%
Age [years]; M (R)			blue laser assisted	6	11.1%
male patients	63 (30–80)		Histopathological findings		
female patients	58 (37–81)		Keratosis	28	51.8%
Lesion site			SIL I	9	16.6%
left VF	19	35.2%	SIL II	3	5.6%
right VF	15	27.7%	SIL III	7	13.0%
both VFs	20	37.1%	others	7	13.0%
Lesion size			Patient treatment response		
≤one VF third	26	48.1%	complete primary resection	42	95.5%
≤two VF thirds	21	38.9%	second look surgery (d/t path)	2	4.5%
entire VF length	7	13.0%	recurrence	6	13.6%
Lesion localization			Patient time course; M (R)		
cranial VF surface	7	13.0%	duration until diagnosis [days]	33 (1–172)	
medial VF edge included	35	64.8%	follow-up period [months]	16 (3–46)	
caudal VF included	12	22.2%	time until recurrence [months]	19 (3–40)	
Phonatory VF mobility (pre)			Phonatory VF mobility (post)		
normal	13	24.1%	normal	41	75.9%
reduced	33	61.1%	reduced	13	24.1%
absent	8	14.8%	absent	0	─

Abbreviations: d/t—due to; M—mean; path—pathology; R—range (lowest value–highest value); SIL—squamous intraepithelial lesion; VF(s)—vocal fold(s).

**Table 2:** Pre- and post-therapeutic voice function parameters (mean ± SD) for all lesions and relevant pathology groups, including mean therapeutic changes (Diff) and 95% confidence intervals (CI) for vocal measure differences three months after leukoplakia removal.

Vocal Measure		Total Group	Keratosis Group	SIL I Group	SIL II + III Group
VEM	Pre Post	81.9 ± 35.8 84.5 ± 32.9	82.1 ± 30.6 78.4 ± 34.6	88.0 ± 24.6 96.3 ± 17.7	59.4 ± 62.1 75.2 ± 33.0
	Diff (CI)	2.6 (−6.9; 12.2)	−3.7 (−21.1; 13.8)	8.3 (−2.1; 18.7)	15.8 (−21.9; 53.5)
DSI	Pre Post	1.8 ± 2.1 1.6 ± 2.3	1.4 ± 2.1 0.8 ± 2.6	1.9 ± 1.8 2.8 ± 1.6	1.3 ± 1.8 1.1 ± 1.9
	Diff (CI)	−0.2 (−0.9; 0.4)	−0.6 (−1.7; 0.5)	0.9 (0.1; 1.7)	−0.2 (−2.9; 2.5)
Jitter (%)	Pre Post	0.8 ± 0.5 0.6 ± 0.3	0.7 ± 0.5 0.6 ± 0.2	0.9 ± 0.5 0.6 ± 0.2	0.8 ± 0.4 0.8 ± 0.6
	Diff (CI)	−0.2 (−0.3; 0.02)	−0.1 (−0.3; 0.1)	−0.3 (−0.8; 0.03)	0.01 (−0.8; 0.8)
MPT (s)	Pre Post	15.4 ± 8.3 18.3 ± 21.6	13.1 ± 7.4 13.3 ± 6.3	17.3 ± 8.0 17.8 ± 9.3	13.5 ± 7.8 17.9 ± 25.6
	Diff (CI)	2.9 (−3.2; 9.1)	0.2 (−3.2; 3.7)	0.5 (−3.2; 4.3)	4.4 (−2.1; 10.9)
VHI-9i	Pre Post	14.3 ± 9.2 9.7 ± 7.7	16.5 ± 9.0 10.7 ± 8.9	10.6 ± 8.0 6.5 ± 6.6	15.2 ± 9.5 12.8 ± 4.5
	Diff (CI)	−4.6 (−7.0; −2.3) ***	−5.8 (−9.1; −2.5) **	−4.1 (−9.2; 0.8)	−2.4 (−13.1; 8.5)
VHIs	Pre Post	1.6 ± 0.9 0.9 ± 0.8	1.6 ± 1.0 0.9 ± 0.9	1.4 ± 0.8 0.7 ± 0.6	1.8 ± 1.0 1.0 ± 0.9
	Diff (CI)	−0.7 (−1.0; −0.4) ***	−0.7 (−1.2; −0.3) *	−0.7 (−1.1; −0.2) *	−0.8 (−2.1; 0.4)
G	Pre Post	1.5 ± 0.7 1.1 ± 0.6	1.5 ± 0.7 1.1 ± 0.6	1.4 ± 0.8 0.9 ± 0.5	1.8 ± 0.8 1.7 ± 0.5
	Diff (CI)	−0.4 (−0.6; −0.2) ***	−0.4 (−0.6; −0.1) **	−0.5 (−1.0; 0.1)	−0.1 (−1.0; 0.6)
R	Pre Post	1.3 ± 0.6 1.1 ± 0.5	1.3 ± 0.7 1.1 ± 0.5	1.3 ± 0.8 0.9 ± 0.5	1.7 ± 0.5 1.7 ± 0.5
	Diff (CI)	−0.2 (−0.5; −0.1) **	−0.2 (−0.5; −0.1) *	−0.4 (−1.0; 0.3)	0.0 (−0.7; 0.7)
B	Pre Post	0.8 ± 0.8 0.6 ± 0.7	0.9 ± 0.8 0.7 ± 0.8	0.6 ± 0.8 0.5 ± 0.7	1.2 ± 1.2 0.7 ± 0.8
	Diff (CI)	−0.2 (−0.4; −0.03) *	−0.2 (−0.4; 0.1)	−0.1 (−0.6; 0.2)	−0.5 (−1.4; 0.4)

Abbreviations: B: breathiness; DSI: dysphonia severity index; G: (overall) grade of hoarseness; MPT: maximum phonation time; R: roughness; VEM: vocal extent measure; VHI-9i: 9-item voice handicap index; VHIs: self-perceived overall vocal impairment. The level of significance is indicated as follows: * significant at *p* < 0.05; ** significant at *p* < 0.01; *** significant at *p* < 0.001 (Wilcoxon signed-rank test).

## Data Availability

The data presented in this study are available on request from the corresponding author. The data are not publicly available due to ethical and privacy restrictions.

## Abbreviations

The following abbreviations are used in this manuscript:

B	Breathiness
CI	Confidence interval
CIS	Carcinoma in situ
CO_2_	Carbon dioxide
DSI	Dysphonia Severity Index
F0	Fundamental frequency
F0max	Maximum fundamental frequency
F0min	Minimum fundamental frequency
G	Overall grade of hoarseness
GRB	Grade, Roughness, Breathiness
HPV	Human papillomavirus
HSIL	High-grade squamous intraepithelial lesion
LSIL	Low-grade squamous intraepithelial lesion
MPT	Maximum phonation time
OR	Odds ratio
R	Roughness
SD	Standard deviation
SIL	Squamous intraepithelial lesion
SPLmin	Soft phonation threshold
VF(s)	Vocal fold(s)
VEM	Vocal Extent Measure
VHI-9i	International 9-item Voice Handicap Index
VHIs	Self-perceived overall vocal impairment
VLS	Videolaryngostroboscopy
VRP	Voice range profile
WHO	World Health Organization

## Appendix A

**Table A1:** VHI-9i questionnaire items (*German translation*) as used in the present study. The original instrument was developed by Nawka et al. [78], and its validation and classification were described in Caffier et al. [40].

Item Text	Score				
My voice makes it difficult for people to hear me. (*Man hört mich wegen meiner Stimme schlecht.*)	0	1	2	3	4
People have difficulty understanding me in a noisy room. (*Anderen fällt es schwer, mich in einem lauten Raum zu verstehen.*)	0	1	2	3	4
The sound of my voice varies throughout the day. (*Der Klang meiner Stimme ändert sich im Laufe des Tages.*)	0	1	2	3	4
My family has difficulty hearing me when I call them throughout the house. (*Meine Familie hört mich kaum, wenn ich zuhause nach ihnen rufe.*)	0	1	2	3	4
My voice difficulties restrict my personal and social life. (*Meine Stimmschwierigkeiten schränken mich in meinem Privatleben ein.*)	0	1	2	3	4
The clarity of my voice is unpredictable. (*Bevor ich spreche, weiß ich nicht, wie klar meine Stimme klingen wird.*)	0	1	2	3	4
My voice is worse in the evening. (*Abends ist meine Stimme schlechter.*)	0	1	2	3	4
I am less outgoing because of my voice problem. (*Ich bin weniger kontaktfreudig wegen meines Stimmproblems.*)	0	1	2	3	4
My voice makes me feel incompetent. (*Wegen meiner Stimme fühle ich mich unfähig.*)	0	1	2	3	4

Scoring: 0 = never (*nie*), 1 = almost never (*selten*), 2 = sometimes (*manchmal*), 3 = almost always (*oft*), 4 = always (immer).

**Table A2:** Global VHIs question added to the study questionnaire.

Question	Score			
How do you rate your voice today? (*Wie schätzen Sie Ihre Stimme heute ein?*)	0	1	2	3

Scoring: 0 = normal (*normal*), 1 = mildly (*leicht*), 2 = moderately (*mittelgradig*), 3 = severely disturbed (*hochgradig gestört*).
